# Training in psychosomatic medicine and psychotherapy for medical doctors in China: A field report

**DOI:** 10.3389/fmed.2023.1119505

**Published:** 2023-04-17

**Authors:** Jing Wei, Kurt Fritzsche, Lili Shi, Jinya Cao, Markus Bassler, Anne-Maria Müller, Ying Zhang, Hannah-Theresa Lüdemann, Rainer Leonhart

**Affiliations:** ^1^Department of Psychological Medicine, Peking Union Medical College Hospital, Peking, China; ^2^Department of Psychosomatic Medicine and Psychotherapy, Faculty of Medicine, Center for Mental Health, Medical Center - University of Freiburg, Freiburg, Germany; ^3^Rehabilitation Sciences and Healthcare Research, Institute for Social Medicine, Mainz, Germany; ^4^Department of General Internal Medicine and Psychosomatics, University Hospital Heidelberg, Heidelberg University, Heidelberg, Germany; ^5^Institute of Psychology, University of Freiburg, Freiburg, Germany

**Keywords:** training, psychosomatic medicine, psychotherapy, curriculum, China, quality assessment, outcome

## Abstract

**Background:**

The high prevalence rates of mental disorders in China contrast a comparatively low care capacity from qualified trained medical doctors in the mental health field. The main objective of our cooperation project was to develop and implement advanced postgraduate training for medical doctors for their acquisition of knowledge, skills, and attitudes in the field of psychosomatic medicine and psychotherapy in China.

**Methods:**

Monitoring and evaluation as part of the advanced training in Beijing were conducted following the Kirkpatrick training approach using four levels of evaluation: reaction, learning, behavior and results. We performed a continuous course evaluation, assessed the respective learning goal attainment, conducted a pre-post evaluation of reasons and goals for participation in the training, and measured the treatment effects on the patient side.

**Results:**

The implementation of training standards in the field of psychosomatic medicine and psychotherapy for medical doctors and the transfer of didactic knowledge and skills for Chinese lecturers were achieved. A total of 142 mainly medical doctors attended the 2-year training. Ten medical doctors were trained as future teachers. All learning goals were reached. The content and didactics of the curriculum were rated with an overall grade of 1.23 (1 = very good to 5 = very bad). The highest rated elements were patient life interviews, orientation on clinical practice and communication skills training. The achievement of learning objectives for each block (depression, anxiety disorders, somatic symptom disorder, coping with physical diseases) was rated between 1 and 2 (1 = very well achieved to 5 = not achieved) for all items from participants’ perspectives. On the patient side (n = 415), emotional distress decreased and quality of life and the doctor–patient alliance improved significantly.

**Discussion:**

Advanced training in psychosomatic medicine and psychotherapy was successfully implemented. The results of the evaluation show high participant satisfaction and the successful achievement of all learning objectives. A more detailed and extensive evaluation of the data, such as an analysis of the development of the participants as psychotherapists, is in preparation. The continuation of the training under Chinese guidance is guaranteed.

## 1. Introduction

Numerous studies have reported that mental disorders have been on the rise in China, as is the case worldwide (1, 2). Concurrent with improved living conditions and improved medical care, China is experiencing a massive rise in mental disorders, which cause high economic and social costs for health care systems (3). Disease burden profiles in China resemble those of Western countries. China’s most recent National Mental Health Survey revealed a weighted 12-month prevalence of any mental disorder of 9·3% and a lifetime prevalence of 16·6% (4). Anxiety and depression disorders were the most common diagnoses. A previous study by Phillips et al. (5) reported even higher 12-month prevalence rates for any mental disorder, reaching up to 17.5% (5). The reasons for the increasing and varying prevalence rates of mental disorders in China have been described (6).

The high prevalence rates for mental disorders contrast a comparatively low care capacity from qualified medical doctors in the mental health field as well as nurses, clinical psychologists and social workers (7). In its country report for China from 2010, the WHO specified a mental health care distribution key of 1.53 psychiatrists per 100,000 inhabitants, with a strong urban–rural gap (7). By the end of 2020, there were more than 50,000 licensed (assistant) psychiatrists nationwide, 4,819 psychotherapists and 40,920 psychological counselors engaged in mental health work in medical institutions (8). In primary health care and general medical care, mental health training and mental health services are scarce. Since the 1980s, the development and professionalization of psychotherapy services has been evident, although intermittent; however, a shortage of systematic training and research remains, and it is imperative to improve the training of medical doctors, e.g., psychiatrists. One decisive step in the right direction was the Mental Health Law, which became effective on May 01, 2013. In 2015, the General Office of China’s State Council published the 2015–2020 National Mental Health Work Plan, which focuses on the recognition and treatment of “severe mental illnesses,” such as schizophrenia, bipolar disorder and mental disability. However, psychosomatic medicine, which refers to the interactions between physical diseases, such as coronary heart diseases, stroke, and diabetes, and common mental disorders, such as depression, are not mentioned, while psychotherapy is referred to only on the periphery (9). Furthermore, the Mental Health Law does not specify which type of treatment is considered psychotherapy and which type of psychotherapeutic training health care professionals have to complete as a prerequisite (10).

From 1949 to the early 1980s, psychotherapy played a small role in the Chinese health care system and in the public. The Chinese were not considered fit to be treated with psychotherapy. Psychotherapy was not “properly and sufficiently applied to Chinese patients by well-trained Chinese psychotherapists” (11). This is in contrast to developments since the early 2000s. Psychological ideas, practices and institutions have gone on to flourish since that time (12). Psychotherapy occupies a central position in the so-called “psycho-boom.” Organizations offer training programs in all current therapy approaches, which is developing into an industry of its own. Nationally and internationally famous psychotherapists spread their psychological knowledge in the form of workshops, books and media-effective public appearances. Private counseling centers and individual private psychotherapy practices are mushrooming, particularly in cities in eastern China. In this way, a new mental health services sector is emerging alongside public psychiatric institutions (13).

The best-known international training program is a Sino-German Course (Zhong de Ban) with three sections (psychoanalysis, cognitive behavioral therapy, and systemic family therapy, including hypnotherapy) (14). Since the 1900s, there have been courses in cognitive-behavioral psychotherapy (CBT), which have also been evaluated (15). A more recent development is the online training of the China American Psychoanalytic Alliance (CAPA) (16–18).

Advanced training courses in psychosomatic medicine and psychotherapy started in 2012 at the Union Hospital in Beijing (19).

Germany has 60 years of experience in the field of psychosomatic medicine and psychotherapy. These years of clinical and scientific experience are well suited to effectively and sustainably support culturally adapted progress in China.

Data on the prevalence of mental disorders in China show that the spectrum of diagnoses is comparable to that in Western countries or Germany, so it is justified to draw on the professional experience and qualification structures from Germany. The German training system has a unique selling point in the field of psychosomatic medicine, which has existed for several decades as an independent specialist discipline with specific indication areas alongside psychiatry. The concepts of psychosomatic medicine and psychotherapy and specialized training in these areas seem to be particularly suitable for the implementation of psychotherapy training according to the Chinese Mental Health Law. There are fundamental differences between the concept of Chinese medicine and the philosophical foundations of Western medicine. These are completely different traditions of philosophy. This must be considered when translating Western medicine into a Chinese context. However, with the biopsychosocial model of psychosomatic medicine, there is already a great approximation to the holistic concept of Chinese medicine. The dualism between body and mind characteristic of Western medicine is overcome. Nevertheless, adjustment processes are necessary with regard to content and didactics.

The authors of this article have been collaborating with Chinese universities and health facilities in various areas of psychosomatic medicine and psychotherapy in recent decades. There have been publications on the development of postgraduate training for medical doctors in psychosomatic medicine (20–23) and reports on the implementation of Balint groups in Chinese hospitals (23).

The evaluation of the current training program had three research questions:

1. How was the training program rated in terms of the content, the didactic methods used, the materials provided, the group atmosphere and the competence and motivation of the trainers?
2. Was there any change in knowledge, skills and behavior of the trainees and how were the new psychosomatic and psychotherapeutic skills transferred and used in daily work?

An additional, secondary question was:

1. Was there any change in the symptoms of patients treated by this type of trained doctors?

If all three research questions are answered positively, this training program can serve as a model project for psychosomatic medicine and psychotherapy training in China and can represent a useful addition to the Chinese medical training system.

## 2. Methods

### 2.1. Training program

#### 2.1.1. Objective of the training

**Figure 1:**
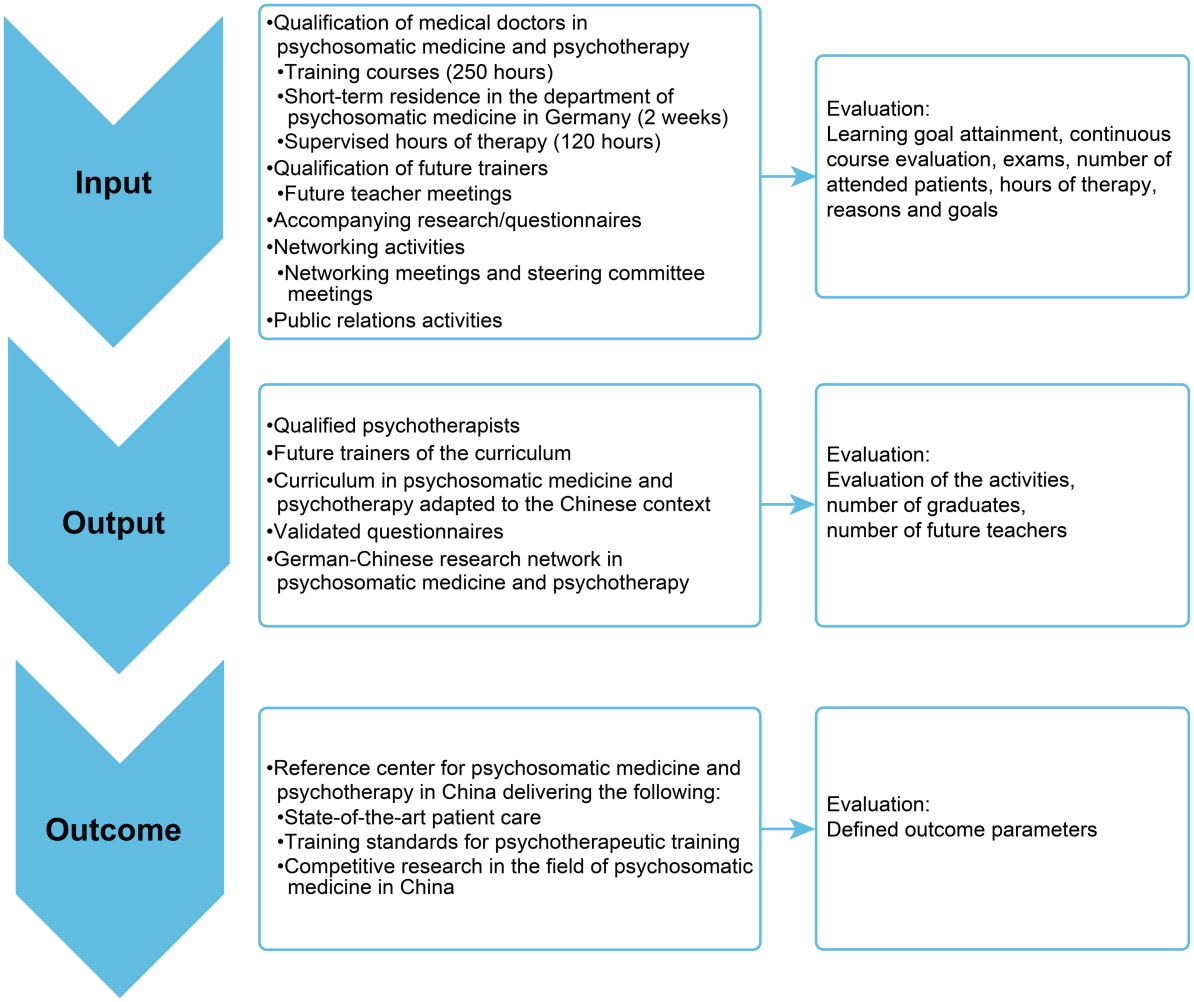
Outputs and outcomes.

The long-term objective of the project is to improve training in psychosomatic medicine and psychotherapy in China and thus improve the mental health care of the population. This project simultaneously provides a curriculum in psychosomatic medicine and psychotherapy adapted to the Chinese context and a framework for psychotherapeutic training and continued education. Activities, outputs and outcomes are summarized in Figure 1. In this article, we report on the content and didactics of the curriculum, the results of the evaluation of two training phases and the effects on patient outcomes.

#### 2.1.2. Curriculum

The main features of the curriculum were previously adapted to the Chinese context in preliminary projects by the University Medical Center Freiburg (20, 21), and they were drawn on in the present project. Characteristics of the curriculum include state-of-the-art scientific and empirically proven teaching methods and techniques and a qualification spanning several schools of therapy based on psychodynamic psychotherapy, supplemented with elements of CBT, systemic family therapy, and hypnotherapy.

The training content were as follows:

- Recognition and differential diagnostic classification of the most common psychological problems and disorders,
- Learning of psychotherapeutic short-term therapies (from 5 to 25 sessions) in individual, couple and family therapy,
- Cooperation with other specialists in the mental health field (psychiatrists, psychologists, counseling centers, and social workers, according to the Mental Health Law) and.
- Learning to teach the curriculum at the professional level of a university department for psychosomatic medicine, i.e., in the sense of the train-of-the-trainer concept.

Training with the German instructors took place in four blocks of 5 days each over the course of 2years. The project had two training cycles. New participants were recruited for the second training cycle. One training cycle included 250 teaching hours in total. There were 4 teaching blocks with each 25 h of theory, 12.5 h self-awareness, and 2.5 h paper case work. That means one block had 40 teaching units. Additionally, in between the teaching blocks the instructors from PUMCH taught 20 h paper case work, 30 h Balint Groups, and 40 h supervision. For details, see Table 1.

**Table 1:** Curriculum overview.

Curriculum	Instructors from Germany	Instructors from PUMCH	Hours
Theory	100 h		100
Self-awareness	50 h		50
Paper case work	10 h	20 h	30
Balint groups		30 h	30
Individual and group supervision of 120 therapy hours		40 h	40
Total			250

From the beginning of the project, future teachers in Beijing were integrated as coteaches. In the second training cycle, they took over the role of teachers under the supervision of the German instructors. This model has already proven successful in the psychosomatic basic care training of Chinese medical doctors in previous projects (21). The language of instruction was English. The curriculum was translated into Chinese. The content of the curriculum and the timetable using the example of depression are summarized in Supplementary material 1 and 2.

#### 2.1.3. Didactics and teaching methods

The didactics aimed for a balance of theoretical knowledge transfer, practical exercises and self-experience. Each topic was enlivened by interactive lectures and exercises. The necessary background knowledge was conveyed in the form of short lectures and materials. Through patient life interviews and role playing, participants experienced the process of a psychotherapy session and gained direct insight into the work and style of the instructors. Therapeutic communication skills and therapeutic relationship building were a common thread through the entire training program. Personal experience and the associated sensitization of the therapist to transference and countertransference processes in everyday professional life had a particular emphasis in the curriculum. Finally, written and oral examinations were conducted. By completing the training blocks and passing the exam, participants could earn a certificate, which was provided by the PUMCH in cooperation with Freiburg University Hospital.

#### 2.1.4. Participants

The main target group of the training program was medical doctors of all specialties, especially psychiatrists. In addition, other professional groups, such as psychologists, nurses and social workers, who work in mental health services, were also welcomed.

#### 2.1.5. Primary and secondary goals

The immediate goals were primarily the establishment of a curriculum of psychosomatic medicine and psychotherapy based on the German 2-year advanced training for medical doctors. Second, the program aspired to transfer didactic knowledge and skills to Chinese lecturers, the so-called “future teachers,” for the independent continuation of the curriculum in the sense of sustainability. The aim was to set training standards for the specialty of psychosomatic medicine and psychotherapy.

### 2.2. Data analysis

Monitoring and evaluation of the advanced training in Beijing were conducted following the Kirkpatrick training approach (24, 25). This framework uses four levels of evaluation (reaction, learning, behavior, and results) based on questions to be answered by different means (see Table 2).

**Table 2:** Evaluation instruments.

	Step 1	Step 2	Step 3	Step 4
	Reactions	Learning	Behavior	Results
Definition	Satisfaction with components, content and organization of teaching	Principles, facts, skills and attitudes understood and absorbed	Use of learned principles or techniques on the job	Outcome parameters
Instrument	- Continuous course evaluation form - Reasons and goals	- Open question in the “Evaluation of training” questionnaire - Goal Attainment Scale (GAS) for each module - Reasons and goals questionnaire at the end of 2-year course - Written multiple choice test at the end of 2-year course - Oral exam	- Open question in the “Evaluation of training” questionnaire - Reasons and goals questionnaire at the end of 2-year course - Orlinsky Psychotherapist Development Questionnaire	- Goal Attainment Scale (GAS). - Reasons and goals questionnaire - Pre-post questionnaires for patients and doctors to evaluate the process and outcome of the treatment sessions - One documented treatment case

#### 2.2.1. Instruments

To evaluate the training in terms of content, didactics, group atmosphere, and motivation of the leaders, an evaluation form with the possibility for open answers was used after each training block.

At block one and after block four, a questionnaire on reasons and goals was used to capture the motivation for specific content and the goals and whether these goals were achieved after 2 years. The achievements of specific learning goals of each block were documented with the Goal Attainment Scale (GAS).

Participants’ professional and personal development were queried using the very comprehensive and detailed Orlinsky Psychotherapist Development Questionnaire (26). The results of the first study phase have been published elsewhere (27).

Each trainee was required to select up to 10 patients who should undergo short-term integrated psychotherapy (ISTP) by himself/ herself during the study period. It was not a random sample. The trainees asked *ad hoc* patients to participate. The sample was selected from all patients treated in the hospital, who had given informed consent.

On the patient side, questionnaires were used for the accompanying evaluation of the psychotherapeutic sessions. Patient data were collected during training and up to 1 year after completion of training. The questionnaires were handed out to each patient by the psychotherapist at the start and the end of the therapy.

The questionnaires used for this purpose included the following:

- HAQ Helping Alliance Questionnaire (quality of the doctor–patient relationship) (28, 29).
- PHQ-15 (Patient Health Questionnaire- Somatic symptom severity) (30).
- PHQ-9 (Patient Health Questionnaire-Depression) (31).
- GAD-7 (Generalized Anxiety Disorder-7) (32).
- SF-12 (12-item Short Form Health Survey; quality of life) (33).

Those are well known and validated questionnaires in China, except the Helping Alliance Questionnaire. The Helping Alliance Questionnaire (HAQ) records characteristics of the therapeutic relationship and can be used in particular for psychotherapy evaluation (process and outcome). It can be used to collect the therapist’s as well as the patient’s perspective. It has 12 items (e.g., “I have the feeling that the therapist understands me,” “I believe that the treatment helps me”) with a 6-point answer format (“1” - “very not applicable” to “6” - “very true”). A total value and two subscale values patient-side relationship satisfaction and patient-side success satisfaction can be calculated. In particular, the success scale is a good predictor for the success of the psychotherapy.

The results should be reported back to therapists and used for quality assurance. For the exam, participants were asked to document one treatment case with the following content: current symptoms, somatic findings, current psychosocial anamnesis, biography, psychodynamic understanding of the symptoms in the framework of the current and past life situation, subjective health beliefs, motivation for psychotherapeutic treatment, treatment goals, psychotherapy plan, course of treatment, outcome and summary.

The authors assert that all procedures contributing to this work comply with the ethical standards of the relevant national and institutional committees on human experimentation and with the 1975 Declaration of Helsinki, as revised in 2008.

#### 2.2.2. Statistical method

For the data analysis, SPSS 27 and R 4.1 were used. For descriptive analyses of the quantitative variables, the mean, standard deviation, and range were calculated. The primary objective of this study was to provide a descriptive description of the results from the various questionnaires administered to training participants and to patients. Statistical analyses were conducted using an alpha level of 0.01 to avoid alpha inflation resulting from multiple tests.

## 3. Results

A total of 142 participants attended at least three training blocks. Due to the organizational and structural framework, not all participants were able to take part in all four blocks. The results of both phases are reported conjointly. *N* = 73–101 completed the questionnaire on somatic symptom disorder, physical illness, anxiety, depression and the questionnaire on reasons and goals. Missing values were not imputed. *N* = 10 participants attended the additional training for future teachers and were available as future teachers for the second training cycle and for the future.

### 3.1. Sociodemographic data

Most participants were female (81.8%). The mean age was 41.32 (SD 8.00). A total of 72.7% were married or living with a partner, and most had one child. A total of 26.0% had a doctoral degree, 29.9% had a master’s degree and 35.1% had a bachelor’s degree. A total of 83.2% were medical doctors or had a medical background (e.g., pharmacology, traditional Chinese medicine). A total of 26.2% of the medical doctors were psychiatrists. The others came from general medicine, neurology, gynecology, oncology, gastroenterology, cardiology, geriatrics and pediatrics. A total of 11.4.% worked as psychological counsellors, and some had a master’s or bachelor’s degree in psychology. A total of 5.4% were nurses. All future teachers were medical doctors. N = 77 participants had previous experience with psychotherapy training. The participants reported training in basic psychosomatic care, including Balint groups, systemic therapy, cognitive behavior therapy, psychoanalytical training, group therapy training, mindfulness training, and others. For most participants, this previous training constituted a short training over a few weekends.

### 3.2. Quantitative evaluation of the curriculum

The items were rated on a scale from 1 = very good to 6 = very poor. In all 4 blocks, the ratings were between 1 and 2. The highest scores for content were given to the patient live interview (1.0), orientation toward clinical practice (1.07), and communication skills training (1.2). For didactic methods, group leader motivation (1.02), consequential and exciting teaching (1.07), the attractive and interesting preparation of issues (1.17), and role play (1.32) were highlighted (see Table 3).

**Table 3:** Evaluation of the content, didactics and organization of the training blocks.

	Item	Depression		Anxiety		Somatic symptom disorder		Physical diseases	
		*N*	*M (SD)*	*N*	*M (SD)*	*N*	*M (SD)*	*N*	*M (SD)*
Group leaders	Motivation of the group leaders	99	1.41 (0.83)	101	1.97 (1.51)	83	1.24 (0.69)	75	1.88 (1.67)
	Consequential and exciting teaching of content	99	1.44 (0.85)	101	2.10 (1.50)	83	1.29 (0.79)	75	1.94 (1.62)
Contents	Issues oriented on clinical practice	100	1.17 (0.51)	101	1.43 (0.96)	83	1.05 (0.21)	75	1.30 (0.75)
	Attractive and interesting clinical practice	99	1.16 (0.53)	101	1.57 (1.12)	83	1.07 (0.26)	75	1.36 (0.88)
	Motivation to work with new learned material	98	1.42 (0.67)	101	1.72 (1.14)	83	1.23 (0.48)	75	1.53 (0.95)
Specific components of teaching	Live interview	100	1.10 (0.52)	101	1.25 (0.89)	83	1 (0)	75	1.15 (0.69)
	PPT lecture	99	1.51 (0.73)	100	1.62 (1.03)	83	1.31 (0.66)	75	1.43 (0.81)
	Communication skills training	100	1.24 (0.57)	101	1.61 (1.11)	83	1.07 (0.26)	75	1.43 (0.82)
	Role play	100	1.25 (0.64)	101	1.44 (0.98)	83	1.10 (0.35)	75	1.30 (0.77)
	Questions and answers	100	1.22 (0.48)	100	1.45 (0.92)	83	1.08 (0.28)	74	1.31 (0.81)
	Written material	100	1.46 (0.72)	100	1.58 (0.99)	83	1.25 (0.56)	75	1.39 (0.78)
	Possibility for discussion and exchange in the group	100	1.31 (0.61)	101	1.55 (1.03)	83	1.16 (0.40)	75	1.27 (0.68)
	Atmosphere in the group	100	1.44 (0.71)	101	1.58 (0.98)	83	1.18 (0.42)	75	1.31 (0.75)
Overall Impression	Overall impression of the course	100	1.17 (0.53)	100	1.45 (1.00)	83	1.06 (0.24)	75	1.22 (0.74)

### 3.3. Achievement of learning objectives in each block

The assessment was made on a scale from 1 = very well achieved to 5 = not achieved. All ratings were between 1 and 2 for all items from participants’ perspectives (see Supplementary material 3). Significant differences between the individual learning objectives in the four blocks were not found. The subjectively very good results could be confirmed by the written and oral examination results. Only five participants did not pass the written or oral exam and thus did not receive the certificate.

### 3.4. Reasons

The results for reasons to participate in the training are shown in Supplementary material 4a. On a scale of 1–6 (1 for not at all, 6 for very true), most scores were approximately 5. Significant differences were not found at a significance level of <0.01. We expect this to be due to a ceiling effect.

### 3.5. Goals

The results for the goals are shown in Supplementary material 4b. The question was asked, “To what extent did the following goals apply to you?” On a scale of 1–6 (1 for not at all, 6 for very true), all scores were between 5 and 6.

Supplementary material 4b shows the goals at the beginning of the training and the extent to which these goals were achieved. Again, on a scale of 1–6 (1 for not at all, 6 for very true), most values were between 5 and 6. Significant differences were not found at a significance level of <0.01. Overall, it appears that all objectives were achieved from the participants’ point of view.

The results on the professional and personal development of the participants of the pilot phase are reported in a separate publication (27). Data collection with a one-year follow-up was completed.

### 3.6. Qualitative evaluation

At the end of the questionnaire, the participants were asked to respond to the following questions: “What have I learned this week?” and “How will it affect my daily work?” The responses to these open written comments were used extensively in the planning of the program (data not shown). In the sense of a formative evaluation, the suggestions for improvement were forwarded to the group leaders and taken into account in the planning of each subsequent block.

In response to the question “What have I learned this week?,” the feedback mainly concerned the following three major areas: (1) diagnosis and treatment of depression, anxiety disorders, somatoform disorders and resource-oriented interventions in physical illnesses with the use of various techniques of psychodynamic psychotherapy, CBT and hypnotherapy; (2) self-growth through the recognition of emotionally basic needs in oneself and in patients; and (3) learning of doctor–patient communication skills (biopsychosocial interview) and shaping of the doctor–patient relationship.

In response to the question “How will it affect my daily work? “, most of the answers concerned better understanding of patients and better interaction or communication, reflection on one’s own role (“I would try to be more aware of my own feelings toward the patient,” “know more about myself”), prevention of burnout and better treatment for patients.

The focus on self-experience and practical aspects during the training was highly appreciated by the participants. Therefore, “constructive feedback” focused on a strong desire to have even more supervision, peer supervision, more practical exercises, more self-experience, more live interviews, smaller groups and further support and guidance by the German teachers.

### 3.7. Patient outcomes

In total, *n* = 415 patients were included in the analysis. The mean number of sessions was 8.95 sessions per patient (SD = 9.10). The mean duration of psychotherapy was 29.10 days (SD = 58.27).

### 3.8. Sociodemographic data of the patients

Sociodemographic data are shown in Supplementary material 5. The mean age of the patients was 36.8 ± 15.34 years. A total of 68.0% of the patients were female, and 30.6% were male. The majority of the patients were located in urban areas (86.5%). Almost half of the patients were married, one-third were single, and the rest were divorced, widowed or other. The educational level in the sample was quite high, with almost two-thirds (61.9%) of the patients having a university degree.

### 3.9. Symptom improvement and quality of life

Table 4 shows the baseline and posttherapy levels of depression, anxiety, severity of physical symptoms, quality of life and patient-rated therapeutic relationship. The results show a significant improvement in all measures, with mostly large effect sizes (*d* > 0.5).

**Table 4:** Patient outcomes.

Questionnaire	Pre *M (SD)*	POST *M (SD)*	Difference *M (SD)*	*t*	*df*	*p*_corr_	*d*
PHQ-15	12.40 (6.14)	5.69 (4.57)	6.71 (5.53)	23.92	388	< 0.001	1.21
PHQ-9	13.31 (6.86)	5.78 (5.09)	7.53 (6.40)	23.21	387	< 0.001	1.18
GAD-7	11.17 (6.30)	4.80 (4.65)	6.37 (6.10)	20.73	387	< 0.001	1.05
SF-12_PCS_	40.87 (9.81)	48.06 (8.15)	7.19 (10.68)	13.72	414	< 0.001	0.67
SF-12_MCS_	40.83 (7.90)	44.42 (7.14)	3.58 (9.41)	7.77	414	< 0.001	0.38
HAQ	26.43 (8.80)	19.81 (6.79)	−6.62 (8.63)	−12.49	264	< 0.001	0.77
HAQ_RS_	12.63 (4.85)	10.02 (3.63)	−2.62 (4.54)	−9.38	264	< 0.001	0.58
HAQ_SUS_	13.80 (4.74)	9.80 (3.57)	−4.01 (4.84)	−13.49	264	< 0.001	0.83

Cases with missing values were excluded pairwise; Patient Health Questionnaire-15 (PHQ-15, total score range from 0 to 30; cut-off points of 5, 10, and 15, representing low, medium, and high somatic symptom severity, respectively); Patient Health Questionnaire-9 (PHQ-9, total score range from 0 to 27; cut-off points of 5, 10, 15, and 20 for mild, moderate, moderately severe, and severe depression, respectively); General Anxiety Disorder Scale (GAD-7, total score range from 0 to 21, with cut-off points of 5, 10, and 15 for mild, moderate, and severe anxiety, respectively); Short Form Health Survey (SF-12, with Physical Component Scale (PCS) and Mental Component Scale (MCS) (total score ranges from 0 to 100, with higher scores indicating a better health-related quality of life); Helping Alliance Questionnaire (HAQ, Scale ranges from 1 = very appropriate to 6 = very inappropriate, HAQRS = Helping Alliance Questionnaire Relationship Satisfaction Subscale, HAQSUS = Helping Alliance Questionnaire Success Satisfaction Subscale); *df* = degree of freedom; *p*_corr_ = Bonferroni adjusted.

### 3.10. Further activities

Other activities to achieve the goals of the project were a 2-week summer school in Freiburg, Germany, a 5-day workshop and symposium on psychosomatic medicine in Beijing, participation in the prestigious annual Palace Forum in Beijing, and the annual 2-week stay of two Chinese physicians from Union Hospital in Beijing as visiting scientists in Freiburg, Germany.

## 4. Discussion

### 4.1. Summary of results

In addition to the implementation of the curriculum, the long-term goal has been to establish a reference center for high-quality patient care in the field of psychosomatic medicine and psychotherapy in China at the Union Hospital in Beijing. Achieving this goal will take several years. The department at Union Hospital in Beijing is a small department but has a dedicated staff. Initial progress has been made in the field of patient care, e.g., more offers of individual and group psychotherapy, and in research, e.g., cooperation projects for the recognition and treatment of somatic symptoms and related disorders (SSRD).

Following the Kirkpatrick training approach (25), in step 1 (“reactions”), participants were very pleased with the program, including the program content, instructional materials, presentation methods, instructors, facilities and organization. All predefined goals were rated highly. This form of evaluation is the most frequently used method but also the least reliable due to the subjectivity of the data obtained (34). Participants are often too polite in their ratings, and a good rating at the end of the training is no guarantee that they will put into practice what they have learned.

Changes in knowledge, skills and behavior (step 2 “learning”) and transfer and use of the new behavior in daily work (step 3 “behavior”) were assessed through the open questions of the training evaluation, through the GAS after each training block, and through the Reason and Goals Questionnaire at the beginning and after 2 years. These data, which again are very subjective, can be interpreted in addition to the objective results of the written multiple-choice test, the documented treatment cases and the oral exam after the 2-year course was completed, which showed a high success rate.

The Reasons and Goals Questionnaire did not show significant results pre- and posttest. We assume this is a ceiling effect. Due to already high scores in the pretest, the posttest could not show a significant improvement.

### 4.2. Patient outcomes

The results of documented patient cases showed a significant improvement in the HAQ, PHQ-15, PHQ-9, GAD-7, and SF-12 scores, with medium to large effect sizes over the course of patient treatment (step 4 “results”). In a metanalysis, the overall short-term effect of cognitive behavioral therapy on Chinese patients’ anxiety and depression was medium in size (15).

The results have to be interpreted with some caution, as we do not have a matched control group design. We did not record possible confounders, such as psychotropic drugs, medical herbs, acupuncture, moxibustion, *taiji quan, qi gong* and other self-healing practices. However, when we look at the participants’ feedback and especially at the open questions, we see that they reported that through the training, their skills in the diagnosis and treatment of mental illnesses improved, as did their abilities in doctor–patient communication and the development of a good doctor–patient relationship. Thus, it stands to reason that we can interpret the very good treatment results as a profound effect of the training on patient treatment.

The therapy dose (9 sessions on average) and the therapy duration (**1** month on average, with a wide range) correspond to the use of psychotherapy in earlier years in China **(**35**)**. Unfortunately, there are no recent comparative data on this topic. However, the therapy dose and the duration of therapy correspond to the concept of short-term therapy, as taught in the course.

### 4.3. Transcultural issues

Moderator analyses showed that the short-term effect of psychotherapy was stronger for culturally adapted cognitive behavioral therapy than for unadopted cognitive behavioral therapy (15). Chinese Taoist cognitive psychotherapy (CTCP) combines elements of cognitive therapy and Taoist philosophy. CTCP has been shown to be an effective and enduring method for the treatment of generalized anxiety disorder patients in urban China (36).

Adaptations to the cultural background of the participants were also necessary in our program. The differences in training between China and Germany concern the following points:

In Germany, the training is part of further training in a medical specialty. After completing the training and an examination, the participants are entitled to bill the health insurance company for psychotherapeutic services. This creates an additional financial motivation. This possibility does not exist in China. The participants receive a certificate that they can submit when applying for a job and that gives them advantages over other applicants in individual cases.

In Germany, strict attention is paid to the attendance of the participants. Missing course content must be made up for. In China, it is difficult to plan long-term appointments, since short-term commitments often arise which prevent continuous participation.

The evaluation of the individual training blocks is better in China than in Germany. On the one hand, this could be due to the already mentioned social desirability in China, but on the other hand it may also be an expression of appreciation for the transfer of interesting knowledge and new skills in the field of psychosomatic medicine and psychotherapy. In Germany, psychosomatic medicine and psychotherapy have long been integrated into medical training and society.

Other transcultural problems concerning confidentiality, the hierarchical doctor–patient relationship, the topic of “breaking bad news,” the handling of negative emotions and language barriers have already been described in detail in a previous training program on psychosomatic basic care for medical doctors (21).

Due to the experience of the German teachers and the use of experienced Chinese teachers, these questions hardly played a role and had little influence on the course of the training.

### 4.4. Comparison to other training programs

The results regarding the evaluation of the curriculum itself are identical to the results of the German curriculum **(**20**)**. Differences exist with regard to implementation in practice. In Germany, after completion of the course and successful examination by the Medical Association, it is possible for every clinically working physician to bill the health insurance for psychotherapeutic services. This increases the motivation to start psychotherapeutic treatments on one’s own as soon as possible. Comparisons with other programs in China and internationally are not possible because of the different target groups and the content.

The standardized training of Chinese psychiatrists is mainly focused on the trainees’ ability to diagnose and treat common mental diseases, mainly focusing on: collecting psychiatric medical history, mental examination, writing psychiatric medical records, diagnosis and differential diagnosis, and using psychiatric drugs. The training requires trainees to know the principles of 2–3 types of psychotherapy (such as psychodynamic psychotherapy, cognitive behavioral psychotherapy), but there is no requirement for the practical ability of psychotherapy. The main difference between our training and standard psychiatric training is that our goal is to improve the trainees’ knowledge and skills of psychotherapy and be able to provide psychotherapy for their patients.

The training of the Sino-German Academy for Psychotherapy (“Zhong de Ban”) focuses on one therapy method (see above). Our basis is the bio-psychosocial model of psychosomatic medicine. Our psychotherapeutic training integrates the three most important therapy methods such as psychodynamic psychotherapy, cognitive behavioral therapy and systemic family therapy. We assume that knowledge and skills in one therapy method are not sufficient to treat all patients successfully.

## 5. Limitations

The participants who fully and successfully completed the curriculum were those with previous experience in psychotherapy and who were highly motivated to treat patients. Therefore, the excellent evaluation results should be interpreted with caution.

No standardized questionnaires were used for training evaluation. The training objectives were too specific and were not reflected by existing evaluation questionnaires.

Because of its subjectivity, the participant’s feedback or “reactions” at the end of each block should also be interpreted with caution. The skills training shows changes within this group. However, there is no control group of participants without skills training. The changes in this study were documented by self-rated questionnaires and not by other variables, with the exception of the results of the written multiple-choice test, the external review of the documented treatment cases and the oral exam.

However, such feedback by self-rated questionnaires is a useful tool. This feedback can reflect a variety of areas, including program content, instructional materials, out-of-class assignments, presentation methods, instructors, facilities, organization and general course evaluation. By using participant feedback, it is possible to obtain a quick reaction at the end of training. The method is easy to administer, tabulate, and analyze.

The final limitation concerns patient selection: The patients were selected by the psychotherapist. This created a bias in relation to the severity of the mental disorder. The trainees can select patients who have moderate problems and with whom they expect a good relationship.

## 6. Conclusion

The goal of developing and implementing a model project for psychosomatic medicine and psychotherapy training in China was achieved. The results of further evaluations will show whether this was associated with an improvement in psychotherapeutic care for patients.

This training program could represent a useful addition to the Chinese medical training system.

This requires further developments in four areas:

1. Implementing this advanced training in psychosomatic medicine and psychotherapy into the standard medical curricula of the Union Hospital in Beijing, including further training of future teachers,
2. Implementing a specialized training curriculum for psychiatric and psychosomatic consultation and liaison services (PPCL) in general hospitals.
3. Supporting the ongoing setup of Departments of Psychiatry and Psychosomatic Medicine in general hospitals.
4. Expanding contacts with stakeholders in the health care and policy communities to integrate the training program within the framework of actions of the Mental Health Act.

## Data availability statement

The original contributions presented in the study are included in the article/Supplementary material, further inquiries can be directed to the corresponding authors.

## Ethics statement

The studies involving human participants were reviewed and approved by Institutional review board of the University of Freiburg registered under the number 155/18 and the institutional review board of Peking Union Medical College Hospital registered under the number S-K276. The patients/participants provided their written informed consent to participate in this study.

## Author contributions

JW was the project leader and responsible for the organization of data collection and helped draft the manuscript. KF made substantial contributions to the conception, design, analysis, and interpretation of the data and drafted the manuscript. LS performed the statistical analyses and prepared the first version of the manuscript. MB made substantial contributions to the study conception and design and was involved as a supervisor. A-MM and YZ made substantial contributions to the study conception, design and data collection. H-TL was involved in drafting the manuscript and revising it critically for important intellectual content. JC participated in the study design and data collection and helped draft the manuscript. RL participated in the study design and performed the statistical analysis. All authors read and improved the final manuscript.

## Funding

MB and KF received a grant awarded by the German academic exchange service (DAAD PAGEL no. 57218137).

## Conflict of interest

The authors declare that the research was conducted in the absence of any commercial or financial relationships that could be construed as a potential conflict of interest.

## Publisher’s note

All claims expressed in this article are solely those of the authors and do not necessarily represent those of their affiliated organizations, or those of the publisher, the editors and the reviewers. Any product that may be evaluated in this article, or claim that may be made by its manufacturer, is not guaranteed or endorsed by the publisher.

## Abbreviations

CBT, Cognitive-behavioral psychotherapy
CAP, China American Psychoanalytic Alliance
PUMCH, Peking Union Medical College Hospital
GAS, Goal Attainment Scale
PHQ-15, Patient Health Questionnaire- Somatic Symptoms Severity
PHQ-9, Patient Health Questionnaire-Depression
GAD-7, Generalized Anxiety Disorder scale
SF-12, 12-item Short Form Health Survey
SSDs, Somatic symptom disorders
HAQ, Helping Alliance Questionnaire
CTCP, Chinese Taoist cognitive psychotherapy
PPCL, Psychiatric and psychosomatic consultation and liaison services
