# Supplementary material for: Training in psychosomatic medicine and psychotherapy for medical doctors in China: A field report
Source: Front Med (Lausanne). 2023 Apr 17;10:1119505. doi: 10.3389/fmed.2023.1119505 (PMC10150650; doi:10.3389/fmed.2023.1119505)
Supplement: Supplementary file 1 [file Data_Sheet_1.PDF]

Supplementary Material

Training in psychosomatic medicine and psychotherapy for medical doctors in China - Concept, implementation and results

Jing Wei, Kurt Fritzsche, Lili Shi, Jinya Cao, Markus Bassler, Anne-Maria Müller, Ying Zhang, Hannah-Theresa Lüdemann, Rainer Leonhart

*** Correspondence:**

Kurt Fritzsche, MD: kurt.fritzsche@uniklinik-freiburg.de

Jing Wei, MD: weijing@pumch.cn

Supplementary Data

1. Curriculum content

Clinical pictures

- Recognition and treatment of anxiety disorders
- Recognition and treatment of depressive disorders
- Recognition and treatment of somatic symptom disorder
- Stress reactions and adjustment disorders in life-threatening physical illnesses (psycho-oncology, psychocardiology)
- Recognition and fundamentals of posttraumatic stress disorder (PTSD)
- Recognition and support of patients with personality disorders, e.g., borderline personality
- Motivational interviewing for patients with addictive disorders
- Recognition and support of patients with eating disorders

Cross-sectional topics

- Developmental psychology including attachment theory
- Transference/countertransference
- Involvement of partners and family
- The diagnostic psychotherapeutic interview
- Psychodynamic diagnostics (relationship, conflict, structure)
- Therapy planning
- Basics of neurobiology of mental disorder
- Psychopharmaceutical treatment

2. Time schedule: Depression

Time	Wednesday Nov. 28	Thursday Nov. 29	Friday Nov. 30	Saturday Nov. 1	Sunday Nov. 2
08.30 – 10.00	Welcome<br><br>Wei Jing, Markus Bassler, Kurt Fritzsche<br><br>Sculpture<br><br>Lecture (1): Development of Psycho-	Lecture (1): Curriculum, objectives and content<br><br>Lecture (6): Symptoms, aetiology and diagnostic classification of depressive disorders	Lecture (9a): Short introduction of Integrative Short-Term Psychotherapy (ISTP)<br><br>Lecture (9):<br><br>Cognitive-behavioural concepts and interventions	Lecture (10):<br><br>Psychodynamic<br><br>concepts<br><br>Lecture (11):<br><br>Psychodynamic-oriented Psychotherapy	Lecture (12):<br><br>Integrative Short-Term Psychotherapy (ISTP)<br><br>for depressive disorders<br><br>-Summary

	somatic Medicine in China<br><br>Zhao Xudong<br><br>Lecture (2): Rules for feedback<br><br>Lecture (3): Patient-live-interview	Lecture (3): Patient live interview	Resource-oriented<br><br>interventions<br><br>Lecture (3): Patient live interview		
10.00 – 12.00	Patient live interview with patient with depressive disorder<br><br>- Genogram<br><br>- Reflecting team<br><br>- Observer groups	Patient live interview with patient with depressive disorder<br>• Genogram<br>• Reflecting team<br>• Observer groups:	Patient live interview with patient with depressive disorder and family<br>• Genogram<br>• Reflecting team<br>• Observer groups:	Group work (6):<br><br>Midwife talk-Formulation of a conflict<br><br>Demonstration of intervention	Group work (8)<br><br>Demonstration of interventions:<br><br>“Depression as the uninvited guest”

12.00 – 14.00	Lunchtime	Lunchtime	Lunchtime	Lunchtime	Lunchtime
14.00 – 15.30	Lecture (4) Pictures of depressive patients<br><br>Body exercise<br><br>Group work (1):<br><br>Depression- Self-awareness<br><br>Lecture (5): Emotional basic needs<br><br>Early childhood experiences:<br><br>Bonding and attachment<br><br>(Film)	Lecture (7): Psychoeducation<br><br>Group work (3):<br>• Recognition depression<br>• Empathy<br>• Psychoeducation “The green prescription”<br><br>Demonstration of intervention	Group work (5): CBT Interventions for depressive disorder<br><br>Demonstration of intervention	Group work (7):<br><br>Working with metaphors<br><br>Demonstration of interventions:<br><br>“Age regression”	Group work continued<br><br>Lecture (13):<br><br>Psychopharmacological treatment of depression disorders ***(Chinese teachers)***

15.30 – 15.45		Break	Break	Break	
15.45 – 17.15	Group work (2):<br><br>Resources and emotional basic needs<br><br>Self-experience in 2 groups:<br><br>Life panorama of<br><br>emotional basic needs	Lecture (8) Suicidality<br><br>Group Work (4): Prevention of suicidality<br><br>Demonstration of intervention<br><br>Self-experience in 2 groups:<br><br>Life panorama	Group work continued<br><br>Self-experience in 2 groups:<br><br>Life panorama	Group work continued<br><br>Self-experience in 2 groups:<br><br>Life panorama	Summary by the lecturer<br><br>Evaluation<br><br>Final round<br><br>Farewell
17.15 – 18.00	Teachers' meeting	Teachers' meeting	Teachers' meeting	Teachers' meeting	Teachers' meeting

3. Evaluation of learning goals for depression, anxiety, somatic symptom disorders, psychosomatics of physical diseases

Depression

Learning Goals	*N*	*M* *(SD)*
Know the criteria (ICD-10) of different depressive disorders and be able to relate them in a diagnostic interview	97	1.42 (.76)
Know the aetiological models of depressive disorders and be able to explain them to patients in your own words	96	1.53 (.79)
Know how to accompany depressive patients with empathy	97	1.39 (.60)
Know the psychoeducation interventions ("green prescription") for activation	96	1.55 (.74)
Know the questions to estimate the risk of suicide	97	1.58 (.75)
Know how to make a nonsuicide contract with the patient	97	1.55 (.71)
Know some cognitive and behavioural interventions	96	1.44 (.68)
Know the basic principles of psychodynamic psychotherapy	97	1.56 (.75)

Notes. *N* = number of participants, *M* = mean, *SD* = standard deviation.

Anxiety

Learning Goals	*N*	*M* (*SD*)
Know the criteria (ICD-10) of different anxiety disorders and be able to relate them in a diagnostic interview	101	1.74 (.93)
Know the biopsychosocial model for anxiety and know how to explore them with a patient	101	1.59 (.85)
Know the vicious circle of anxiety and be able to apply it to the patient	100	1.52 (.85)
Know the aetiological models of anxiety disorders and be able to explain them to patients in your own words	100	1.64 (.85)
Know how to establish a therapeutic relationship with patients with anxiety disorders	101	1.56 (.84)
Know elements of integrated short-term psychotherapy (ISTP) including psychodynamic and cognitive-behavioural interventions and be able to apply them to a patient with anxiety disorders	100	1.73 (.92)

Know the different emotional basic needs and know how to apply them to a patient	101	1.56 (.84)
Know about your own biographical issues of anxiety and bee able to note them in a consultation	101	1.72 (.87)
Know the psychotropic drugs in anxiety disorders and use them appropriately	99	1.79 (.97)

Notes. N = number of participants, *M* = mean, *SD* = standard deviation.

Somatic Symptoms Disorders

Learning Goals	*N*	*M* *(SD)*
Know the diagnostic criteria of somatic symptom disorders	83	1.36 (.65)
Know the diagnostic criteria of conversation disorder	83	1.42 (.73)
Be able to practice “feeling understood” (Step 1) in patients with somatic symptom disorders	83	1.53 (.75)
Be able to practice “making the link” between physical symptoms and psychosocial problems in patients with somatic symptom disorders (Step 2)	83	1.66 (.86)
Know how to treat patients with chronic pain	83	1.77 (.86)

Know more about own body reactions in stressful situations	83	1.41 (.64)
Know how to use metaphors to help the patient to gain an alternative understanding of his symptoms	83	1.63 (.82)
Know how to deal with difficult patients	83	1.49 (.67)
Be able to practice handling aggressive/demanding patients	83	1.96 (.90)

Notes. *N* = number of participants, *M* = mean, *SD* = standard deviation.

Psychosomatics of Physical Diseases

Learning Goals	*N*	*M* *(SD)*
Know the psychosomatic aspects of cancer	74	1.27 (.62)
Know the coping strategies of cancer patients	74	1.34 (.65)
Know how to break bad news	74	1.33 (.64)
Know the aims of consultation/liaison interventions	74	1.33 (.60)

Know some resource-oriented interventions	74	1.23 (.61)
Know why and how to involve the partner/family	74	1.32 (.57)
Be able to practice a family interview	74	1.70 (.78)
Individualized psychotherapy: know the why and how of choosing and making a specific intervention	75	1.63 (.86)
Know what burnout is	75	1.34 (.68)
Know how to prevent burnout	74	1.41 (.68)
Know your inner drivers and know how to moderate them	75	1.43 (.72)

Notes. *N* = number of participants, *M* = mean, *SD* = standard deviation.

4. Reasons and goals

4a. Reasons for participation

Reasons for participation	*N*	Pre *M (SD)*	*N*	Post *M (SD)*
Acquisition of knowledge of psychosomatic medicine and psychotherapy	73	5.74 (0.55)	71	5.32 (1.49)
Curiosity about Western methods of psychosomatic medicine and psychotherapy	73	5.47 (0.82)	73	5.12 (1.48)
A desire to get to know the historical development of psychosomatic medicine and psychotherapy	72	5.08 (1.16)	73	4.81 (1.53)
A hope to get to know myself better	72	5.35 (0.97)	73	5.11 (1.42)
A hope to promote my professional career	73	5.84 (0.41)	73	5.26 (1.52)
A desire to experience interesting case studies	73	5.51 (0.73)	73	5.16 (1.46)
A desire to improve English language skills at work	72	4.26 (1.39)	72	4.00 (1.64)
A desire to get to know my personal strengths and weaknesses	72	5.04 (1.17)	73	4.58 (1.69)
A hope to learn to speak more easily in public	72	4.11 (1.71)	72	4.22 (1.77)

Reasons for participation	*N*	Pre<br>*M (SD)*	*N*	Post<br>*M (SD)*
Training as a particular personal challenge	72	4.38<br>(1.60)	72	4.51<br>(1.76)
A hope to improve communication skills	72	4.82<br>(1.47)	73	4.74<br>(1.67)

Notes. Information is based on observed data from participants who have participated in at least 3 blocks; *N* = number of participants, *M* = mean, *SD* = standard deviation.

4b. Goals for participation

Goals for participation	*N*	Pre<br>*M (SD)*	*N*	Post<br>*M (SD)*
Get to know diagnostics in psychosomatic medicine and psychotherapy	70	5.27<br>(0.96)	73	4.93<br>(1.36)
Improve understanding of the history and the behaviour of psychosomatic patients	71	5.52<br>(0.73)	73	5.04<br>(1.93)
Improve understanding of the doctor–patient relationship in psychosomatic patients	71	5.54<br>(0.71)	73	5.11<br>(1.44)
Improve communication skills with psychosomatic patients	71	5.45<br>(0.77)	72	4.99<br>(1.35)

Goals for participation	*N*	Pre *M (SD)*	*N*	Post *M (SD)*
Increase job satisfaction in dealing with psychosomatic patients	71	5.38 (0.80)	73	4.93 (1.40)
Improve opportunities for the design of the doctor–patient relationship	71	5.42 (0.77)	73	5.14 (1.41)
Learn about interesting case studies	71	5.11 (0.93)	73	4.96 (1.35)
Learn how to plan psychotherapy	71	5.14 (1.00)	73	4.71 (1.36)
Learn how to link somatic and psychosomatic/psychotherapeutic goals in therapy	71	5.38 (0.88)	73	4.95 (1.36)
Learn how to recognize transfer issues and how to deal with them	71	5.14 (1.05)	73	4.55 (1.26)
Learn how to assign better psychotherapeutic approaches to the elements of psychodynamic cognitive-behavioural or systemic therapies	71	5.28 (0.99)	73	4.71 (1.37)
Learn how to improve communication skills at work	71	5.34 (0.89)	73	4.89 (1.37)
Learn how to understand people better regarding their feelings and motivations	71	5.30 (0.93)	73	4.88 (1.37)
Learn how to improve one's own teaching methods	71	4.79 (1.21)	73	4.16 (1.36)

Goals for participation	*N*	Pre *M (SD)*	*N*	Post *M (SD)*
Learn how to identify burdensome emotional and mental disorders	70	5.30 (0.87)	73	4.97 (1.39)
Learn how to integrate somatic and psychotherapeutic treatment	71	5.38 (0.80)	73	4.97 (1.42)
Learn how to motivate patients for psychotherapy when it appears indicated	71	5.18 (0.96)	73	4.70 (1.38)
Learn how to perform psychotherapeutic interventions	71	5.17 (1.07)	73	4.67 (1.36)
Learn how to evaluate the effects of one's own psychotherapeutic interventions	71	4.99 (1.06)	73	4.44 (1.26)
Learn where my strengths are in dealing with patients	70	4.87 (1.12)	73	4.55 (1.30)
Learn where my weaknesses/issues are in dealing with patients	70	4.96 (1.11)	73	4.60 (1.30)

Notes. Information is based on observed data from participants who have participated in at least 3 blocks; *N* = number of participants, *M* = mean, *SD* = standard deviation.

5. Sociodemographic characteristics of patients

Variable	N = 415
Age	36.8 ± 15.34
Sex	
Male	127 (30.6 %)
Female	282 (68.0 %)
Area of residence	
Urban	359 (86.5 %)
Rural	45 (10.8 %)
Marital status	
Single	158 (38.1 %)
Married	203 (48.9 %)
Divorced	19 (4.6 %)
Widowed	10 (2.4 %)
Other	17 (4.1 %)
Employment status	
Employed	183 (44.1 %)
Unemployed	30 (7.2 %)
Retired	38 (9.2 %)
Housewife	27 (6.5 %)

Variable	N = 415
Student	119 (28.7 %)
Other	12 (2.9 %)
Educational level	
Primary school	14 (3.4 %)
Junior middle school	48 (11.6 %)
Senior middle school	85 (20.5 %)
University degree or higher	257 (61.9 %)
